# Supplementary material for: A tissue-specific self-interacting chromatin domain forms independently of enhancer-promoter interactions
Source: Nat Commun. 2018 Sep 21;9:3849. doi: 10.1038/s41467-018-06248-4 (PMC6155075; doi:10.1038/s41467-018-06248-4)
Supplement: Supplementary file 1 — Supplementary Information [file 41467_2018_6248_MOESM1_ESM.pdf]

A tissue-specific self-interacting chromatin domain forms independently of enhancer-promoter interactions

Brown et al

SUPPLEMENTARY INFORMATION

Supplementary Figure 1

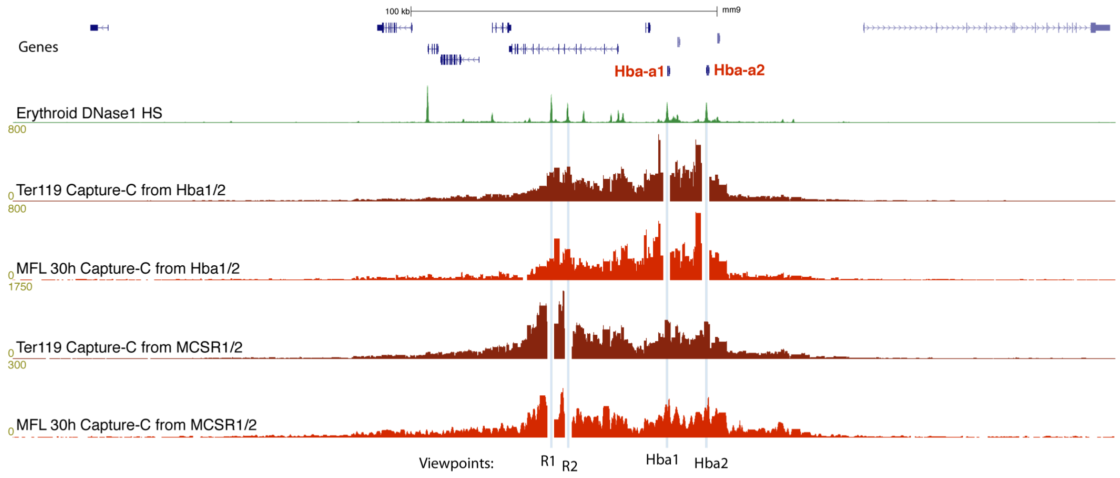

Supplementary Figure 1: Erythroblasts derived from adult spleen or foetal liver form the same domain of chromatin interactions at the α-globin locus. Layout as for Fig. 1e. NG Capture-C tracks from *Hba1/2* and MCSR1/R2 viewpoints show the same pattern of chromatin interactions between erythroblasts derived from adult spleen (Ter119+ dark red) and foetal liver (MFL 30h red).

Supplementary Figure 2

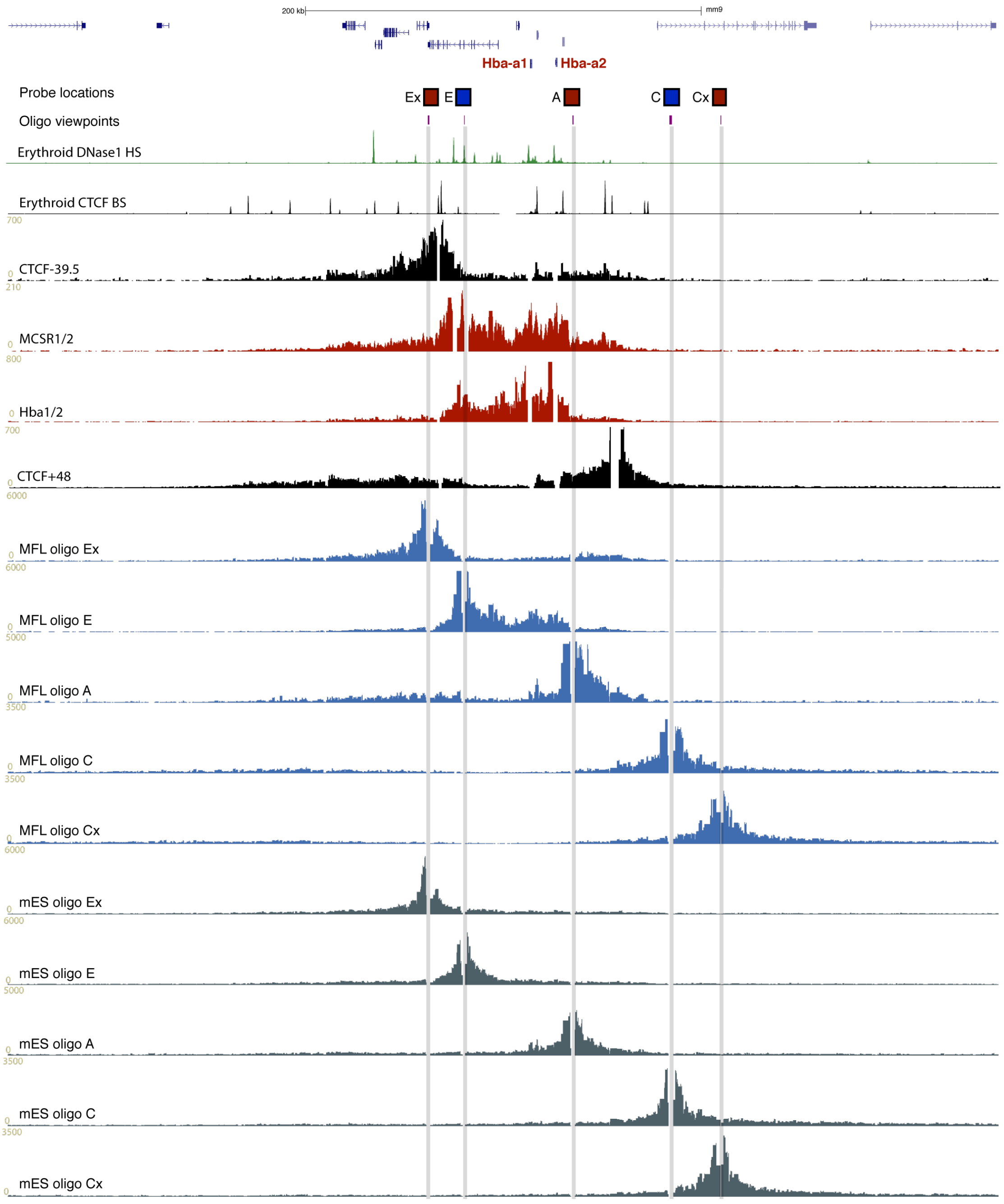

Supplementary Figure 2: Chromatin interactions detected from FISH probe viewpoints in erythroblasts and mES cells. *Hba* genes are highlighted in red, followed by the locations of the plasmid (Ex, E, A, C, Cx) FISH probes and the 50mer oligonucleotides used for capture. Underneath are genome browser tracks showing DNase1 HS (green) and CTCF BS (black), then NG Capture-C tracks for MFL 30h from viewpoints CTCF BS -39.5 (black), the two major enhancer elements MCS-R1/R2, and the *Hba1/2* genes (both red) and the CTCF BS +48 (black). Below are five NG Capture-C

tracks from MFL 30h erythroblasts (blue) depicting interactions from the viewpoints of the five FISH probes, as indicated. Interactions detected by oligo Ex mirror those detected from the CTCF BS -39.5 at the upstream side of the α-globin domain; oligo E detects interactions within the self-interacting domain, matching the Enhancer R1/R2 track; oligo A detects interactions at the opposite side of the domain whilst control oligos C and Cx principally detect proximity interactions. Measurements A to E will therefore reflect a mixture of interactions within and across the domain. Five further tracks (grey) depict interactions in E14 mES cells where only proximity interactions are detected.

Supplementary Figure 3

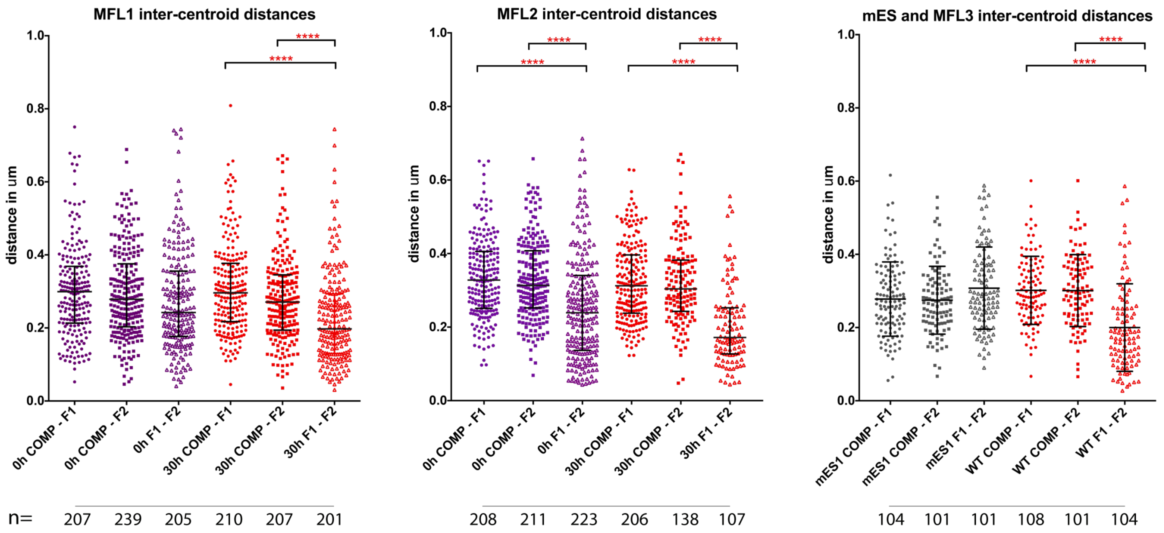

Supplementary Figure 3: Proximity measurements across a 320 kb region encompassing the α-globin locus in mouse WT cells. Inter-centroid distance measurements between three BAC probe pairs, COMP-F1 (circles), COMP-F2 (squares) and F1-F2 (open triangles) in erythroblasts derived from three independent MFL cultures and in mES cells (grey). MFL1 and MFL2 measurements are given for two time points, 0h (purple) and 30h (red). Each dot represents a single measurement and the total number of measurements is indicated by ‘n’. Error bars indicate median value and interquartile range. Statistical significance of differences in range of measurements, derived by a Kruskal-Wallis test with Dunn’s multiple comparisons, is shown (****$p<0.0001$).

Supplementary Figure 4

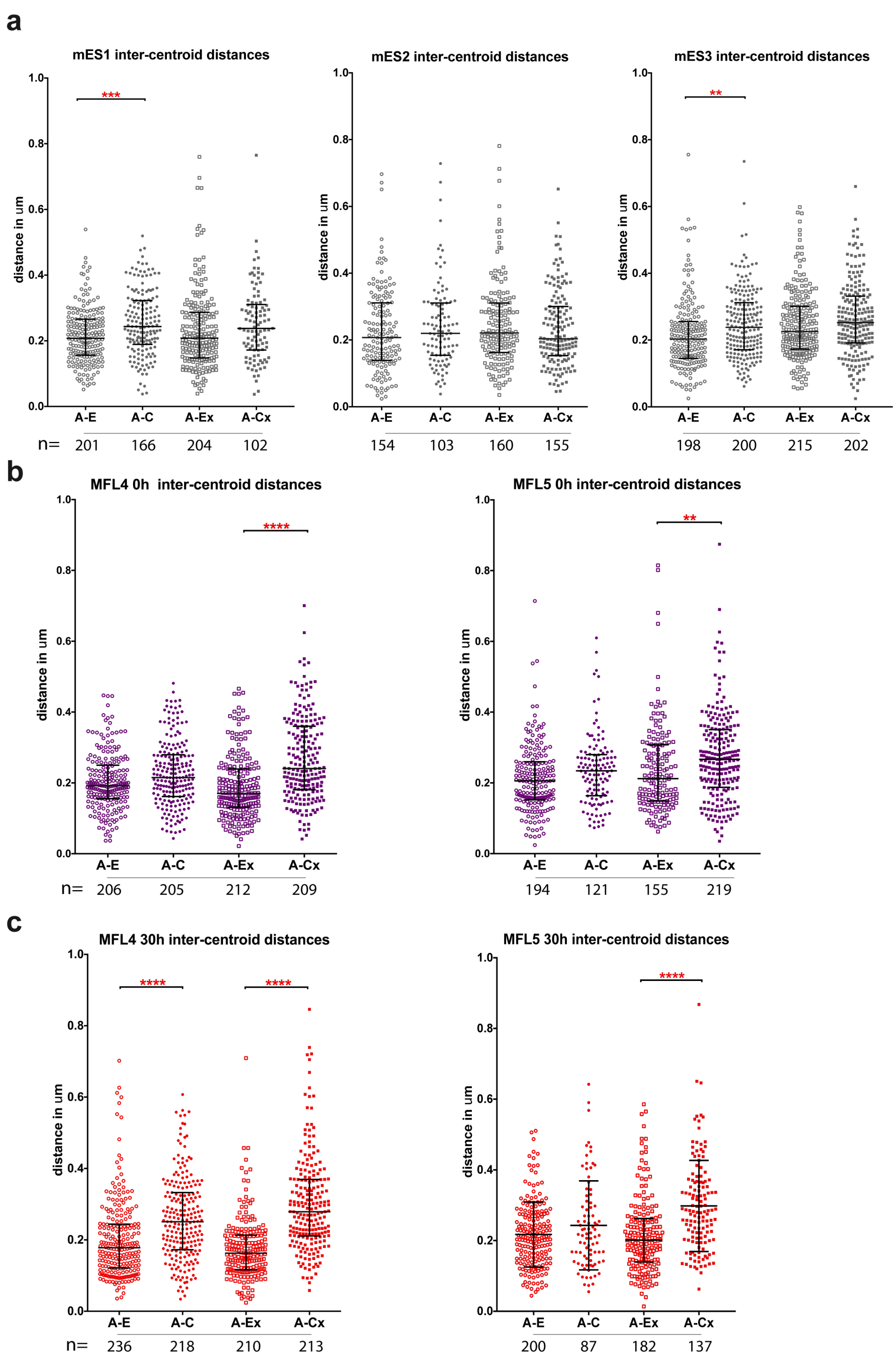

Supplementary Figure 4: Proximity measurements at the α-globin locus between plasmid probe pairs. **a,** Inter-centroid distances measured between the four probe pairs A-E (open circles), A-C (closed circles), A-Ex (open squares), A-Cx (closed squares) in three mES cell cultures (grey). Each dot represents a single measurement and the total number of measurements is indicated by 'n'. Error bars indicate median value and interquartile range. Statistical significance of differences in range of measurements, derived by a Kruskal-Wallis test with Dunn's multiple comparisons, is shown. **b,** Inter-centroid distances plotted as above for two MFL cultures harvested at 0h (purple). **c,** Inter-centroid distances plotted as above for two MFL cultures harvested at 30h (red). ****$p < 0.0001$; ***$p < 0.001$; **$p < 0.01$.

Supplementary Figure 5

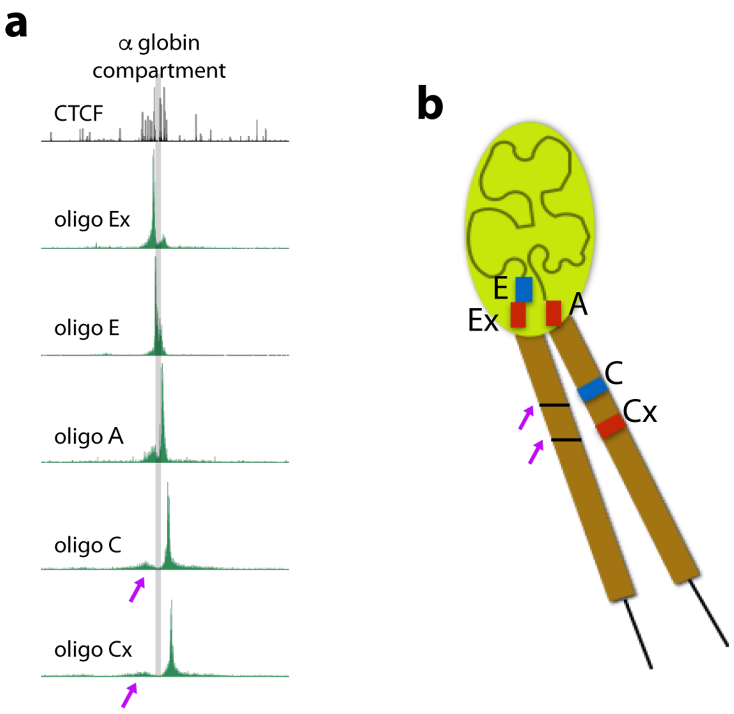

Supplementary Figure 5: Infrequent interactions occur between chromatin regions encompassing the α-globin self-interacting domain. (a) A larger scale view of NG Capture-C tracks for MFL 30h from plasmid probe viewpoints as presented in Fig. S2. The pale grey bar defines the extent of the α-globin domain. Magenta arrows indicate the outlying interactions detected by oligo C and Cx with a region devoid of genes or erythroid-specific accessibility. Careful examination indicates that C interacts rather more frequently and with a region that is slightly closer than Cx. Such interactions are consistent with the development of a distinct domain that affects the positioning of the flanking regions. **(b)** Schematic model of the domain showing that the structure created by the self-interacting domain can lead to more frequent interactions between surrounding chromatin.

Supplementary Figure 6

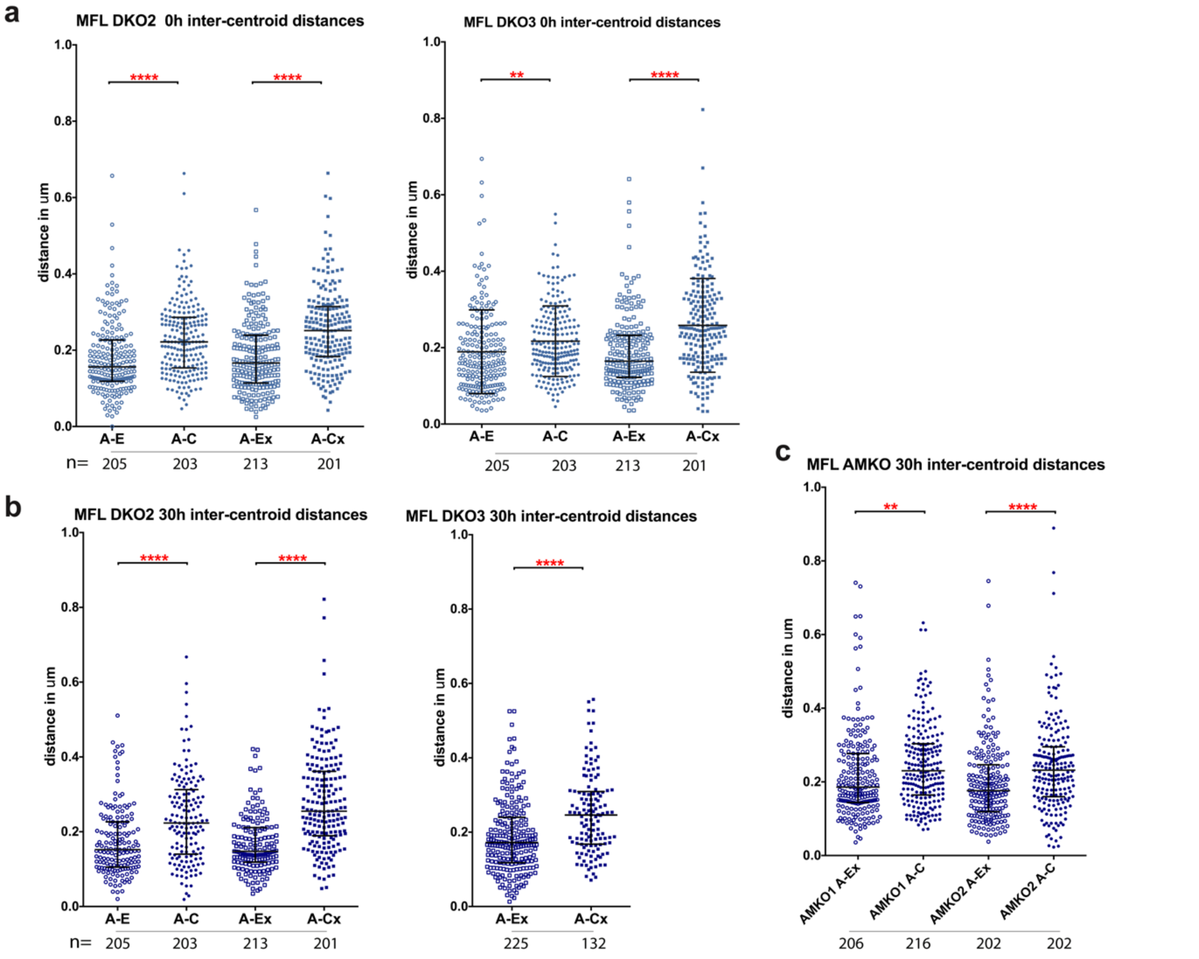

Supplementary Figure 6: Proximity measurements at the α-globin locus between plasmid probe pairs in knockout mouse lines. (a) Inter-centroid distances measured between the four probe pairs A-E (open circles), A-C (closed circles), A-Ex (open squares), A-Cx (closed squares) in two MFL DKO erythroblast cultures at 0h (light blue). Each dot represents a single measurement and the total number of measurements is indicated by 'n'. Error bars indicate median value and interquartile range. Statistical significance of differences in range of measurements, derived by a Kruskal-Wallis test with Dunn's multiple comparisons, is shown. **(b)** Inter-centroid distances plotted as above for two MFL DKO cultures harvested at 30h (dark blue). **(c)** Inter-centroid distances plotted as above for two MFL AMKO cultures harvested at 30h (dark blue). ****p< 0.0001; ***p< 0.001; **p< 0.01.

Supplementary Figure 7

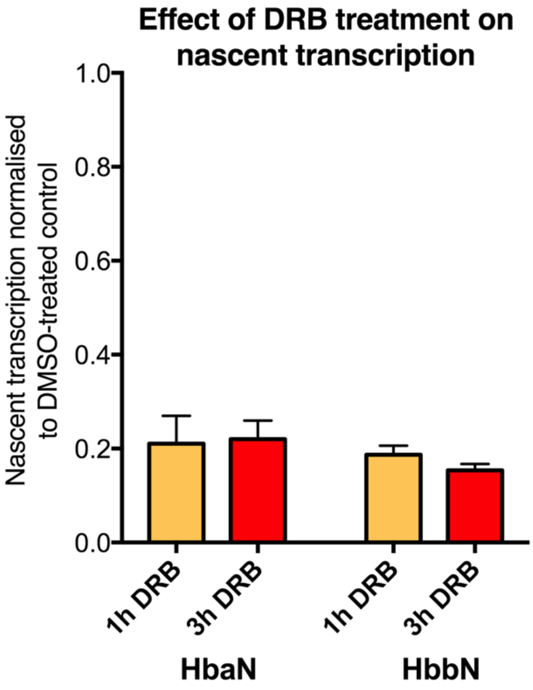

Supplementary Figure 7: Validation of reduction of transcription by DRB treatment. The effect of 1 h or 3 h DRB treatment upon nascent transcription (denoted N) was measured by RT-qPCR of *Hba* and *Hbb*, using *Rn18s* as the control target. The resulting reduction in transcription is shown relative to the vehicle only (DMSO) treated cells. The graph shows the average of technical triplicates, with variation shown as s.d.

Supplementary Figure 8

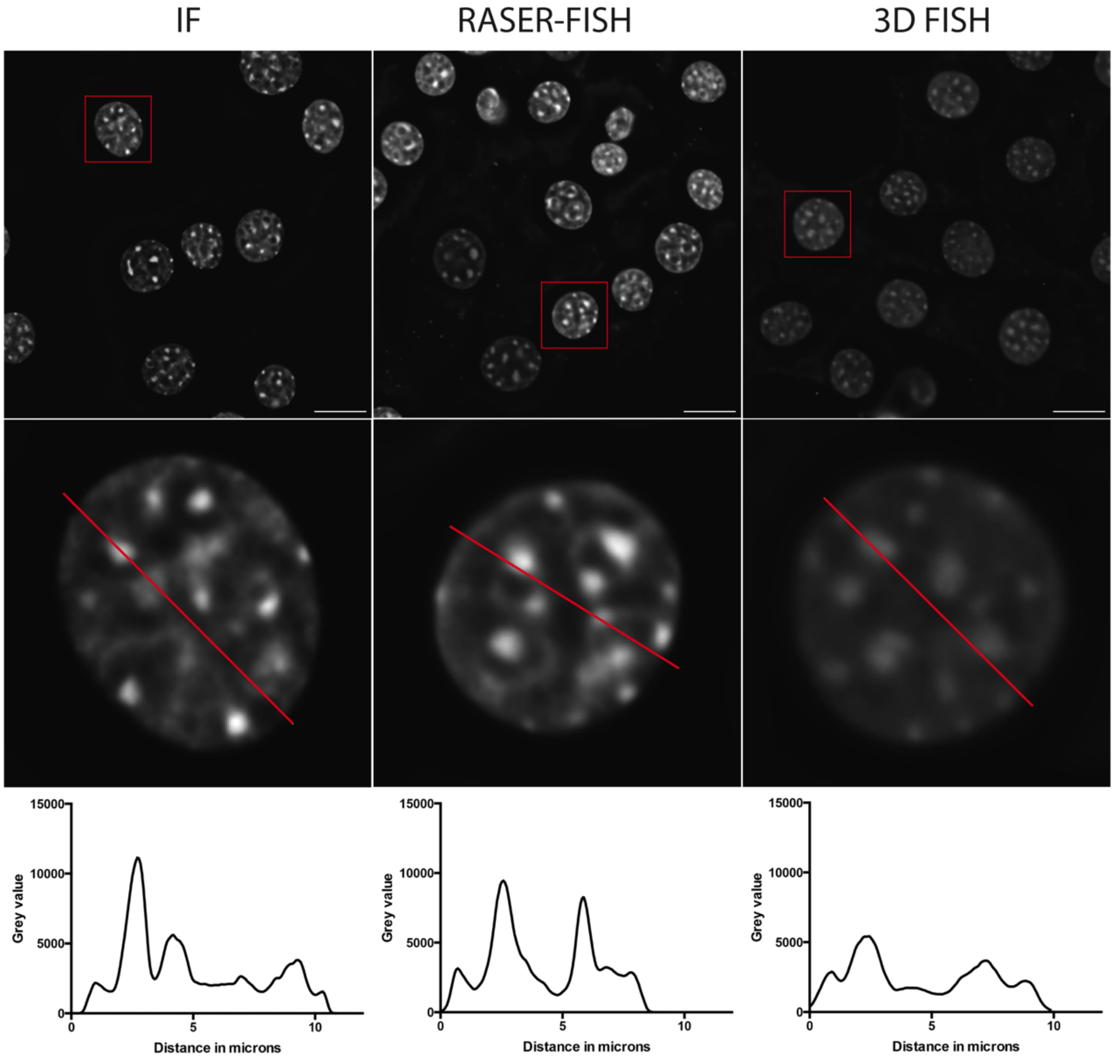

Supplementary Figure 8: The RASER-FISH technique. Example C127 DAPI-stained nuclei (top) after fixation and immunofluorescence only (left), RASER-FISH (middle) or 3D-FISH (right). Scale bar 10μm. Red box indicates selected nucleus; red line across these nuclei (middle) indicates position of line profiles (bottom) indicating fluorescence intensity across the matching nucleus, reduced after 3D-FISH.

Supplementary Figure 9

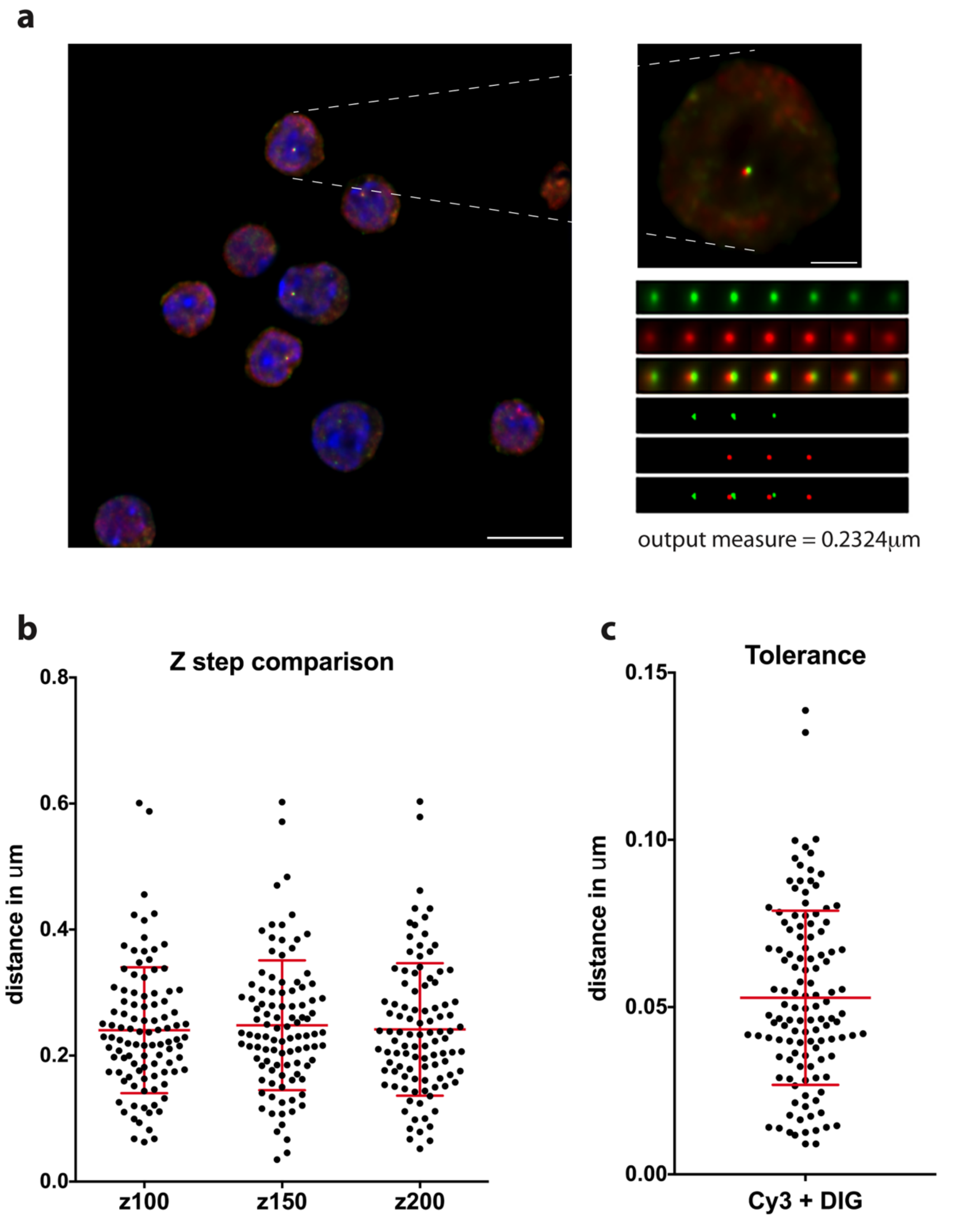

Supplementary Figure 9: Image capture and analysis. **(a)** Example field of capture after deconvolution (left) with selected nucleus (right), above the sub-stack of 7 Z steps generated from the RASER-FISH signal. The signal is thresholded at 80% of maximum fluorescence then distance between signal centroids is automatically calculated in three dimensions after chromatic shift correction. Scale bars 10 μm and 2 μm. **(b)** A-C distance measurements from the same signal pairs were taken after collection of the image stacks at three different Z steps; 100, 150 and 200 nm. There is no difference between the spread of data or mean values for the three data sets. n=101. **(c)** The tolerance of a FISH experiment represents the distance that can be measured between different fluorescent labels to the same probe. Here we hybridised two pools of oligos for MCS-R2 directly labelled with Cy3 and digoxigenin and find a mean tolerance of 53 nm, well below measurements across the α-globin locus. n=121.
